# Mechanism Underlying Defective Interferon Gamma-Induced IDO Expression in Non-obese Diabetic Mouse Fibroblasts

**DOI:** 10.1371/journal.pone.0037747

**Published:** 2012-05-25

**Authors:** Azadeh Hosseini-Tabatabaei, Reza Baradar Jalili, Yunyuan Li, Ruhangiz T. Kilani, Alireza Moeen Rezakhanlou, Aziz Ghahary

**Affiliations:** Department of Surgery, University of British Columbia, Vancouver, British Columbia, Canada; University of Michigan Medical School, United States of America

## Abstract

Indoleamine 2,3-dioxygenase (IDO) can locally suppress T cell-mediated immune responses. It has been shown that defective self-tolerance in early prediabetic female non-obese diabetic (NOD) mice can be attributed to the impaired interferon-gamma (IFN-γ)- induced IDO expression in dendritic cells of these animals. As IFN-γ can induce IDO in both dendritic cells and fibroblasts, we asked the question of whether there exists a similar defect in IFN-γ-induced IDO expression in NOD mice dermal fibroblasts. To this end, we examined the effect of IFN-γ on expression of IDO and its enzymatic activity in NOD dermal fibroblasts. The results showed that fibroblasts from either prediabetic (8 wks of age) female or male, and diabetic female or male (12 and 24 wks of age respectively) NOD mice failed to express IDO in response to IFN-γ treatment. To find underlying mechanisms, we scrutinized the IFN- γ signaling pathway and investigated expression of other IFN-γ-modulated factors including major histocompatibility complex class I (MHC-I) and type I collagen (COL-I). The findings revealed a defect of signal transducer and activator of transcription 1 (STAT1) phosphorylation in NOD cells relative to that of controls. Furthermore, we found an increase in MHC-I and suppression of COL-I expression in fibroblasts from both NOD and control mice following IFN-γ treatment; indicating that the impaired response to IFN-γ in NOD fibroblasts is specific to IDO gene. Finally, we showed that an IFN-γ-independent IDO expression pathway i.e. lipopolysaccharide (LPS)-mediated-c-Jun kinase is operative in NOD mice fibroblast. In conclusion, the findings of this study for the first time indicate that IFN-γ fails to induce IDO expression in NOD dermal fibroblasts; this may partially be due to defective STAT1 phosphorylation in IFN-γ-induced-IDO signaling pathway.

## Introduction

Indoleamine 2,3-dioxygenease (IDO), a rate limiting intracellular enzyme, is involved in metabolism of tryptophan in the biosynthesis pathway of nicotinamide adenine dinucleotide [1]. The importance of IDO in induction of self-tolerance and feto-maternal tolerance during pregnancy in mice has been well documented [1], [2]. It is well established that IDO plays a substantial role in modulation of T cell-mediated immune responses. IDO-induced local tryptophan depletion and accumulation of kynurenine (Kyn), a tryptophan metabolite, can both contribute to the suppression of T cell-mediated immune response [2]–[8].

Since the discovery of immunolegulatory characteristics of IDO, this enzyme has been shown to have far-ranging roles in mammalian pregnancy [2], [5], autoimmune diseases [1], [9], inflammation and allergy [10], neoplasia [11], [12] and most recently inhibition of alloimmune responses in transplantation [13], [14]. Our research group has also shown that skin grafts survival is improved in the presence of IDO-expressing-fibroblasts [15]–[20].

Grohmann and colleagues [9] have shown that tolerogenic property of CD8^+^ dendritic cells (DCs), mediated by IDO expression, is impaired in early prediabetic non-obese diabetic (NOD) mouse strain. They found that interferon-γ (IFN-γ), a potent inducer of IDO expression, selectively fails to induce tryptophan catabolism in CD8^+^ DCs of early prediabetic female NOD mice. They suggested that temporary blockade of a renowned IFN-γ signaling pathway, Janus kinase/signal transducer and activator of transcription 1 (JAK/STAT1), caused by IFN-γ-induced-peroxynitrite generation, explains this phenomenon. As IFN-γ is a potent IDO inducer in many different cell types including fibroblasts, here, we asked the question of whether there exists a similar defect in IFN-γ-induced IDO expression in dermal fibroblasts from NOD mice and if so, whether this is limited to pre-diabetic female mice. To address this, we investigated the IFN-γ-induced-IDO expression and tryptophan catabolism in dermal fibroblasts of non-diabetic (C57BL/6 mice used as control group), prediabetic (8 weeks of age) male and female, diabetic female (12 weeks of age) and male (24 weeks of age) NOD mice. The findings showed that in contrast to C57BL/6 fibroblasts, NOD dermal fibroblasts from either prediabetic or diabetic animals, regardless of their gender failed to express IDO in response to IFN-γ treatment. Further analysis revealed a defect in STAT1 phosphorylation as the potential underlying mechanism of this phenomenon.

## Results

### IFN-γ Fails to Induce Tryptophan Catabolism and IDO Expression in Splenic DCs and Dermal Fibroblasts of NOD Mice

**Figure 1:**
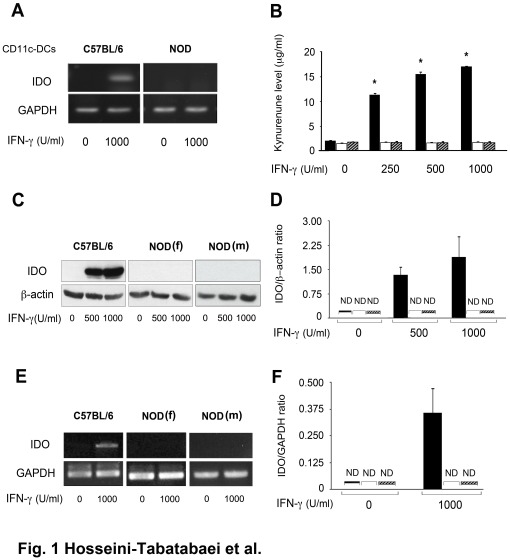
Effect of IFN-γ on IDO expression in splenic DCs and dermal fibroblasts of NOD mice. Tryptophan catabolism and IDO expression at the protein and mRNA levels in cultured splenic CD11c^+^ DCs and fibroblasts from non-diabetic male C57BL/6 mice (solid bars), diabetic female NOD mice at 12 weeks of age (open bars), and diabetic male NOD mice (hatched bars) at 24 weeks of age following IFN-γ treatment. Splenic DCs were treated with 1000 U/ml of IFN-γ or left untreated for 24 hours. Skin fibroblasts from both mouse strains were treated with increasing doses of IFN-γ (0, 250, 500, 1000 U/ml) for 48 hours. **A**: IDO mRNA expression in the treated splenic DCs, **B**: Kyn level in CM of the cells was measured as an indicator of IDO activity. **C**: IDO expression at the protein level, **E**: IDO expression at mRNA level. **D** and **F**: the Mean±SEM ratio of densities of IDO to β-actin at the protein and mRNA levels, respectively. β-actin and GAPDH were used as loading controls for western blotting and RT-PCR respectively. *corresponds significant difference between C57BL/6 and NOD fibroblasts treated with same concentration of IFN-γ (n = 5, p<0.001). ND: not detected.

To confirm the previous findings by Grohmann et al. [9], splenic DCs were isolated from diabetic female NOD (>12 weeks of age) and C57BL/6 mice and treated with 1000 U/ml of IFN-γ or left untreated for 24 hours. Cell pellets were subjected to RT-PCR analysis for evaluation of IDO mRNA expression (Figure 1A). Consistent with previous investigation, our result showed that in contrary to C57BL/6 splenic DCs, IFN-γ failed to induce IDO mRNA expression in NOD splenic DCs. Next, we tend to examine the IDO activity and tryptophan catabolism in dermal fibroblasts from non-diabetic C57BL/6 (male, 12 weeks of age) and diabetic NOD (female, 12 weeks of age; male, 24 weeks of age) mice. Fibroblasts were treated with different doses of IFN-γ for 48 hours. The conditioned media (CM) of the cells were then subjected to Kyn measurement. The result shown in Figure 1B indicates a dose dependent increase (11.35±1.78, 15.52±1.69 and 17.05±1.81 µg/ml respectively) (P<0.01, n = 6) in Kyn level in CM from various concentrations of IFN-γ-treated C57BL/6 cells relative to that of untreated control (2.08±1.09). However, there was no increase in Kyn levels produced by IFN-γ-treated female NOD fibroblasts (1.78±0.09, 1.69±0.09 and 1.81±0.08 µg/ml respectively) or male NOD fibroblasts (1.81±0.095, 1.72±0.08, 1.78±0.085 compared to that of non-treated ones (1.57±0.05 and 1.85±0.03 µg/ml for female and male NOD fibroblasts respectively); suggesting that no active IDO proteins were present in NOD fibroblasts. To further investigate at which level (i.e. mRNA production or protein expression) this error occurred, fibroblast RNA and cell lysates were subjected to RT-PCR and western blot analysis, respectively. The results showed that, similar to the enzymatic activity, IFN-γ failed to induce IDO expression at the protein (Figure 1C and 1D) and mRNA (Figure 1E and 1F) levels in NOD dermal fibroblasts. Figures 1D and 1F represent the quantitative analysis of the data shown in Figures 1C and 1E, respectively. IDO protein and mRNA were remarkably expressed in C57BL/6 cells in a dose-dependent manner in response to IFN-γ treatment (P<0.01, n = 6). These data suggest that IFN-γ-mediated-tryptophan catabolism is impaired in dermal fibroblasts of NOD mice.

**Figure 2:**
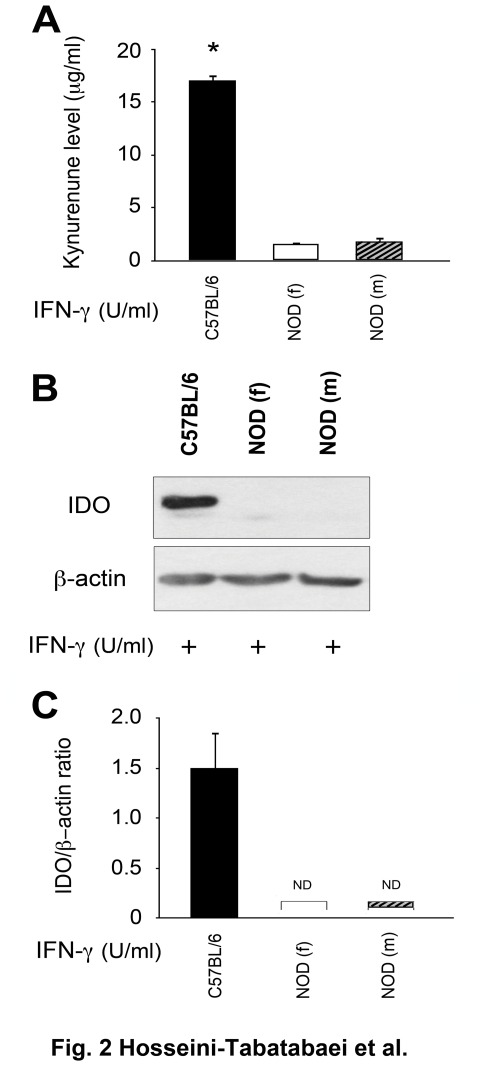
Different effect of IFN-γ on IDO expression in dermal fibroblasts of C57BL/6 prediabetic NOD mice. Dermal fibroblasts from prediabetic (8 weeks of age) male and female NOD mice failed to respond to IFN-γ induced IDO. Dermal fibroblasts isolated from C57BL/6 male mice of 8 weeks of age as control (solid bars), and aged matched male (hatched bars) or female (open bars) prediabetic NOD mice were treated with 1000 U/ml of IFN-γ for 48 hours. **A**: Kyn levels in CM of treated cells, **B**: IDO expression at the protein level, **C**: the Mean±SEM ratio of densities of IDO to β-actin at protein control group treated with IFN-γ (n = 3, p<0.01). β-actin expression showed equal loading of proteins. ND: not detected.

To examine the effect of gender and diabetes status on IDO induction by IFN-γ, dermal fibroblasts isolated from prediabetic male and female NOD mice (8 week of age) and control male C57BL/6 mice (8 weeks of age) were treated with IFN-γ for 48 hours. The level of Kyn, as an index for IDO activity, was measured and our result showed that in contrary to C57BL/6 cells, fibroblasts from neither male nor female prediabetic NOD mice were able to catabolize tryptophan to Kyn (Kyn levels 17.05±0.28 µg/ml for C57BL/6 cells versus 1.68±0.22 and 1.71±0.14 µg/ml for female and male prediabetic NOD fibroblasts respectively) (Figure 2A). Comparing fibroblasts from female and male prediabetic NOD with control cells, we found that regardless of gender, dermal fibroblasts from 8 weeks old prediabetic NOD mice fail to express IDO following IFN-γ treatment at the protein level. However, C57BL/6 cells significantly expressed active IDO (P<0.01, n = 6) (Figure 2B). The quantitative analysis of the blots was also consistent with this result (Figure 2C).

### IFN-γ Can Induce MHC-I Expression in Dermal Fibroblasts of NOD Mice

**Figure 3:**
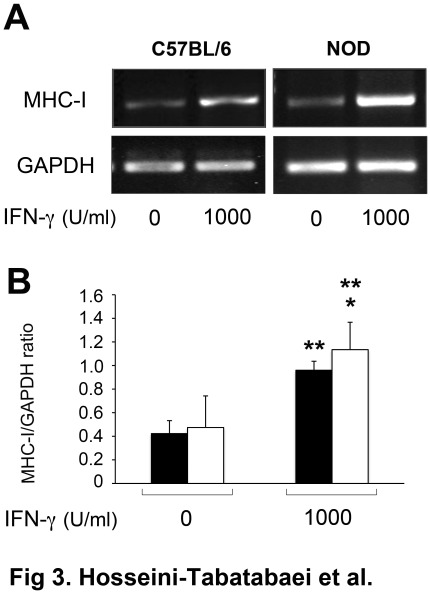
MHC-I mRNA expression in fibroblasts isolated from control and NOD mice. Cells were treated with 0 or 1000 U/ml of IFN-γ for 48 hours. **A**: RT-PCR analysis of MHC-I mRNA expression. **B**: the Mean±SEM ratio of densities of MHC-I to GAPDH. Solid and open bars represent C57BL/6 and NOD fibroblasts respectively. GAPDH was used as loading control. *denotes significant difference between C57BL/6 and NOD fibroblasts treated with IFN-γ in terms of MHC-I expression (n = 3, p<0.05). **corresponds to significant difference between cells from the same strain treated with 0 or 1000 U/ml of IFN-γ (n = 3, p<0.01).

In order to get insights into the mechanism underlying impaired responsiveness of NOD fibroblasts to IFN-γ, we first asked whether the expression of other IFN-γ-mediated genes is also defective in these cells. As a well-established molecule upregulated by IFN-γ [21], [22], we evaluated the major histocompatibility complex class I (MHC-I) mRNA expression using RT-PCR in dermal fibroblasts of 12 weeks old C57BL/6 (male) and diabetic NOD (female) mice following treatment with IFN-γ. Activation of JAK/STAT1 signaling pathway has been previously shown to be involved in IFN-γ-mediated MHC-I upregulation [22]. In addition to this, there are compelling evidences suggesting the role of nuclear factor-kappaB (NF-κB) pathway in the regulation of MHC class I gene expression [23]. Therefore, any defect in MHC-I expression through JAK/STAT1 pathway, can be compensated through the activity of NF-κB pathway. As shown in Figure 3A, IFN-γ significantly increased MHC-I mRNA expression in both C57BL/6 and NOD fibroblasts compared to that of untreated ones (P<0.05, n = 6). The quantitative analysis of these signals revealed a significant difference in MHC-I level between IFN-γ treated NOD fibroblasts and that of untreated control (P<0.05, n = 6) (Figure 3B). This data shows that IFN-γ can bind to its receptor at the surface of NOD fibroblasts and stimulates the expression of MHC-I. As such the defect seen in IFN-γ-induced-IDO expression in NOD fibroblasts is likely to be specific to the signaling pathway of IDO expression.

### IFN-γ Reduces Collagen Expression in Dermal Fibroblasts of NOD Mice

**Figure 4:**
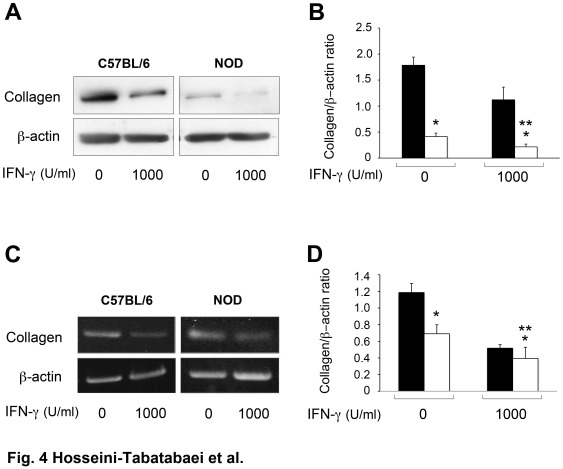
COL-I expression in dermal fibroblasts from control and NOD mice. COL-I expression in dermal fibroblasts from C57BL/6 (solid bars) and NOD (open bars) mice was evaluated by western blot and RT-PCR analyses. Cells were exposed to 0 or 1000 U/ml of IFN-γ for 48 hours before analysis. **A**: COL-1 expression at the protein level. **C**: COL-1 expression at mRNA level. **B** and **D** represent the Mean±SEM ratio of COL-1 to β-actin at protein and mRNA levels respectively. β-actin was used as loading control in both western blotting and RT-PCR assays. *demonstrates significant difference between C57BL/6 and NOD fibroblasts treated with IFN-γ in terms of COL-1 expression. **corresponds to significant difference between cells from the same strain treated with 0 or 1000 U/ml of IFN-γ (n = 3, p<0.05).

It is well documented that IFN-γ reduces the expression of collagen through activity of CCAAT/enhancer-binding protein β (C/EBPβ), a JAK/STAT1-independent mediator [24]. To further investigate the responsiveness of other genes to IFN-γ, fibroblasts from 12 weeks old male C57BL/6 and diabetic female NOD fibroblasts were treated with IFN-γ or left untreated for 48 hours. The result showed a significant difference in type I collagen (COL-1) expression between untreated and treated NOD fibroblasts at the protein level (P<0.05, n = 6) (Figure 4A). Interestingly, the quantitative analysis showed that the expression of COL-1 in untreated NOD fibroblasts was significantly lower compared to that of C57BL/6 fibroblasts (P<0.01, n = 6) (Figure 4B). Consistent with the protein expression, the result of RT-PCR showed that IFN-γ was capable of suppressing COL-1 mRNA expression significantly (P<0.05, n = 6) in both treated C57BL/6 and NOD fibroblasts compared to untreated cells (Figure 4C). The quantitative analysis of RT-PCR assay is shown in Figure 4D.

Taken together, these results show that IFN-γ receptor and other IFN-γ mediated-protein expression pathways seem to be functional. This further confirms that the defect is specific for IFN-γ-induced-IDO expression pathway in dermal fibroblasts of NOD mice.

### IDO Gene-transduced NOD Fibroblasts Express IDO

**Figure 5:**
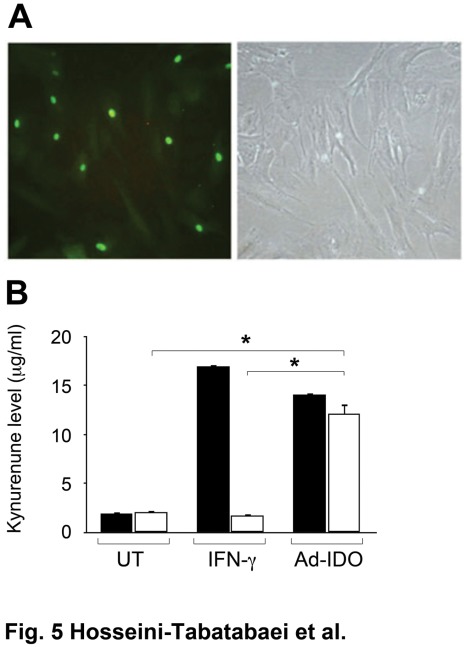
Tryptophan catabolism and microscopic evaluation of IDO expression in dermal fibroblasts following transduction with Ad-IDO vector. Fibroblasts were treated with either blank medium or IFN-γ (1000 U/ml), or transduced with Ad-IDO vector carrying GFP at an MOI of 100. A: IDO and GFP expression in transfected fibroblasts. B: Increased levels of Kyn indicate that NOD (open bars) and C57BL/6 (solid bars) fibroblasts express active IDO following IFN-γ (48 h post-treatment) or Ad-IDO treatment (72 h-post transfection). *denotes significant difference between related bars. (n = 3, p<0.001). Data are reported as Mean±SEM. UT: untreated.

To gain in-depth perspective on the mechanism underlying impaired IFN-γ-induced tryptophan catabolism in NOD dermal fibroblasts, we next asked whether the ribosomal protein production machinery is intact in these cells. In other words, we investigated whether the NOD fibroblasts are capable of translating IDO mRNA to functional IDO protein. To achieve this, we employed an adenoviral vector bearing IDO and green florescent protein (GFP) reporter genes (Ad-IDO) to transduce C57BL/6 (12 weeks of age, male mice) and diabetic NOD (12 weeks of age, female mice) dermal fibroblasts as described previously [18]. A mock vector was used as control for IDO bearing vector. A separate batch of fibroblasts from both NOD and C57BL/6 mice were also treated with 1000 U/ml of IFN-γ. Microscopic evaluation of transfected NOD fibroblasts after 72 hours, confirmed the viability of the cells and successful transfection as indicated by GFP expression under UV light (Figure 5A). To investigate the efficacy of IDO transfection in fibroblasts, conditioned media (CM) of the cells treated with IFN-γ or transduced with Ad-IDO were collected for Kyn measurement. The result showed a significant increase (P<0.001) in Kyn levels in CM of Ad-IDO transduced NOD (12.13±0.94 µg/ml) and C57BL/6 (14.09±0.10 µg/ml) fibroblasts compared to untreated cells (2.09±0.14 and 1.94±0.22 for NOD and C57BL/6 fibroblasts respectively) suggesting the functionality of expressed IDO in these cells. In contrary to NOD fibroblasts (Kyn level 1.74±0.19 µg/ml), C57BL/6 fibroblasts were able to catabolize tryptophan following IFN-γ treatment markedly (Kyn level 16.97±0.10 µg/ml) (P<0.001, n = 6). (Figure 5B).

**Figure 6:**
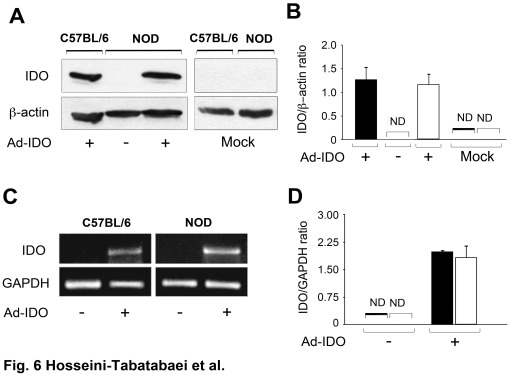
IDO protein and mRNA expression in Ad-IDO transfected cells. Dermal fibroblasts from C57BL/6 (solid bars) and NOD (open bars) mice were transduced with Ad-IDO or mock vector. **A**: IDO expression was analyzed by western blotting, **C**: IDO expression was analyzed by RT-PCR. **B** and **D**: the Mean±SEM ratio of IDO to β-actin at the protein and GAPDH at mRNA level (n = 3). β-actin and GAPDH were used as a loading control for protein and mRNA expression respectively. ND: not detected.

For assessing IDO expression at protein and mRNA level, the Ad-IDO transduced cells were harvested and both protein and mRNA expressions were evaluated by western blot analysis and RT-PCR, respectively. The results confirmed a high level of IDO expression at protein level in Ad-IDO transduced NOD and C57BL/6 cells relative to vector treated cells (Figure 6A). The quantitative analysis of the data is shown in Figure 6B (P<0.001, n = 6). Consistent with this, the result of RT-PCR analysis showed that Ad-IDO transduced NOD and C57BL/6 fibroblasts markedly expressed IDO mRNA (Figure 6C). The quantitative analysis of this data is shown in Figure 6D (P<0.001, n = 6). Collectively, these results elucidate that the unresponsiveness of NOD fibroblasts to IFN-γ treatment in terms of IDO expression is not due to a defect in translation of the IDO mRNA to IDO protein. As such these findings suggest that this defect is likely to be at the IFN-γ-induced-IDO specific upstream signaling pathway.

### Defective STAT1 Phosphorylation is Responsible for Impaired IFN-γ-induced Tryptophan Catabolism in NOD Fibroblasts

**Figure 7:**
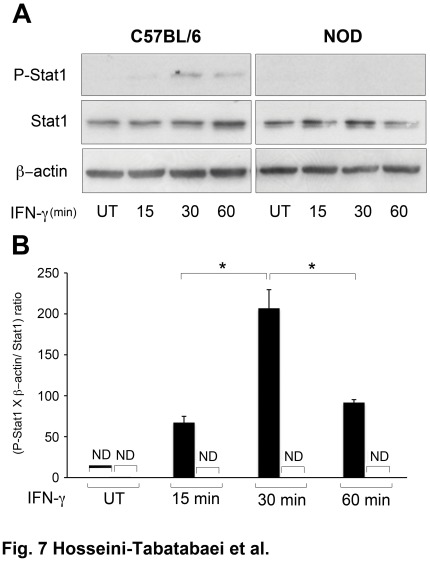
IFN-γ-induced-STAT1 phosphorylation in C57BL/6 and NOD dermal fibroblasts. Following starvation for 18 hours, dermal fibroblasts from NOD (open bars) and C57BL/6 (solid bars) mice were remained untreated or treated with 1000 U IFN-γ per ml of DMEM plus 2% FBS for 15, 30 or 60 minutes. Cell lysates were collected for western blot analysis. **A**: STAT 1 phosphorylation shown by western blotting. **B**: the Mean±SEM ratio of phospho-STAT1 (P-STAT1), to the ratio of β-actin to total STAT1. Total STAT1 and β-actin expressions were used as loading controls. *denotes significant difference between related bars (p<0.05, n = 3). UT: untreated, ND: not detected.

It is well established that IFN-γ activates phosphorylation of JAK and subsequently STAT1. The phosphorylated STAT1 then translocates to the nucleus and initiates transcription of responding genes [25], [26]. Knowing this, we monitored the STAT1 phosphorylation in diabetic female NOD and male C57BL/6 dermal fibroblasts following treatment with IFN-γ at different time points (0, 15, 30 or 60 minutes). The result showed that following treatment, IFN-γ induced STAT1 phosphorylation in C57BL/6 fibroblasts with a peak at 30 minutes. However, it failed to induce STAT1 phosphorylation in NOD fibroblasts (Figure 7A) (P<0.01, n = 6). The quantitative analysis of this data is shown in Figure 7B. The expression of phosphorylated STAT1 was normalized to the expressions of total STAT1 and β-actin. This finding indicates that a defect in STAT1 phosphorylation should, at least in part, be responsible for IFN-γ failure to induce IDO expression in these cells.

### LPS Induces IDO Expression in NOD Dermal Fibroblasts

**Figure 8:**
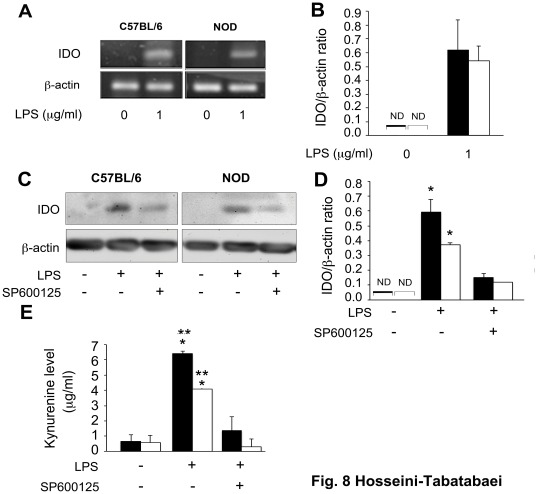
LPS-induced IDO expression in C57BL/6 and NOD dermal fibroblasts. In panels A and B, C57BL/6 (solid bars) and NOD (open bars) were treated with 0 or 1 µg of LPS from *Pseudomonas aeruginosa* per ml of DMEM plus 2% FBS, for 24 hours. **A**: RT-PCR analysis of IDO mRNA expression. **B**: the Mean±SEM ratio of densities of IDO to β-actin. GAPDH was used as a loading control. In panels C, D and E, cells were treated with 1 g/ml of LPS in the presence or absence of SP600125 (10 M), a JNK inhibitor, for 48 hours. C: western blot analysis of IDO expression, D: the Mean±SEM ratio of densities of IDO to β-actin. **E**: The Kyn levels indicate that LPS induced the expression of active IDO enzyme in NOD and C57BL/6 fibroblasts, which was mediated through JNK pathway. *denotes significant difference between cells from the same strain treated with LPS with or without SP600125 (p<0.05, n = 3). **represents significant difference between LPS-treated and non-treated cells from the same strain (P<0.001, n = 3). ND: not detected.

Using another IDO inducer, we next examined whether the expression of IDO via an IFN-γ-independent pathways is also defective or not. To investigate this, C57BL/6 and NOD (12 weeks of age, male and female respectively) dermal fibroblasts were treated with 1 µg/ml of lipopolysaccharide (LPS), derived from *Pseudomonas aeruginosa* [27] for 24 hours. Using primary murine microglia, it has been previously shown that LPS can induce IDO expression via an IFN-γ-independent mechanism with a pick of enzymatic activity at 24 hours. This mechanism depends upon activation of the c-Jun-N-terminal kinase (JNK) pathway [28]. Our result showed that LPS was able to induce IDO mRNA expression in both C57BL/6 and NOD dermal fibroblasts (P<0.05, n = 3) (Figure 8A). Figure 8B represents the quantitative analysis of this data. Next, the control and test cells were treated with 1 µg/ml of LPS in the presence or absence of SP600125 (10 µM), a JNK inhibitor, for 48 hours. The result of western blot analysis showed that both C57BL/6 and diabetic NOD fibroblasts markedly (P<0.05, n = 3) expressed IDO following treatment with LPS. This effect was partially reversed in the presence of the JNK inhibitor (Figure 8C). The quantitative analysis of the data is shown in Figure 8D. The result of Kyn assay was in parallel with the protein assay and the data showed that both groups were able to significantly catabolize tryptophan, while SP00125 partially reversed this effect (Figure 8E). Again, this finding suggests that a defect in STAT1 phosphorylation is likely to be responsible for IFN-γ failure to induce IDO expression in NOD fibroblasts.

## Discussion

Knowing that IFN-γ-induced IDO expression in many different cell types including fibroblasts is important in controlling the number and viability of CD4^+^ and CD8^+^ T cells [29], here we asked the question of whether IFN-γ induces the expression of IDO in fibroblasts from an autoimmune diabetic mouse model. We compared dermal fibroblasts from NOD and C57BL/6 mice in terms of: 1) IFN-γ-induced IDO expression and enzymatic activity, 2) Other IFN-γ-mediated pathways such as MHC-I expression, 3) Modulation of COL-I expression, 4) IDO protein translational capacity, 5) Phosphorylation of STAT1, a key signal transducer in IFN-γ-mediated-IDO expression pathway and finally, 6) Ability of NOD cells to express IDO in response to an IFN-γ-independent stimulus such as LPS.

Our results showed that IFN-γ fails to induce IDO expression in dermal fibroblasts of NOD mice. The NOD strain of mouse is a renowned animal model of autoimmune diabetes. This strain develops a spontaneous T cell-mediated destruction of pancreatic beta cells with a higher incidence in females compared to males. The onset of diabetes is about at 12 to 14 weeks of age in females and shortly after in male NOD mice [30]. In this study, we evaluated the effect of IFN-γ in expression and enzymatic activity of IDO in a selection of NOD mice; diabetic (female, 12 weeks of age; male, 24 weeks of age) and prediabetic (8 weeks of age, female or male) NOD mice were examined. The rationale for this selection was the previous observation in tolerogeneic DCs, suggesting that only DCs from early prediabetic female NOD mice fail to express IDO following IFN-γ treatment. The findings revealed that differentially from DCs, dermal fibroblasts from none of these groups expressed IDO and as a result no enzyme activity was found as evaluated by measurement of Kyn level.

Considering the fact that type I diabetes is developed by massive infiltration of T cells in pancreatic islets and expression of IDO have a T cell suppressive effect [29], it would be important to understand how IDO expression is involved in controlling the pancreatic inflammation. Structurally, surrounds pancreatic islets, there exists natural scaffold produced by fibroblasts [31]. Fibroblasts play a major role in improving the survival and functionality of islets. Here we hypothesized that failure of these fibroblasts to express IDO might also play a role in suppressing autoreactive T cells invading pancreatic beta cells. It is likely that IFN-γ released from activated T cells induces IDO expression in fibroblasts leading to a balance between tolerance and immune response. Therefore, the findings of our study showing a defect in tryptophan catabolism in diabetic fibroblasts along with those of DCs can be partially involved in impaired tolerance towards autoreactive T cells.

MHC-I molecule is an abundant protein found at the cell surface of all nucleated cells. In fact these molecules play an important role in initiation of T cell mediated events [32]. This study showed that IFN-γ is capable of inducing MHC-I in NOD dermal fibroblasts. Moreover, COL-1 levels in cell lysates were measured following IFN-γ treatment. We found that IFN-γ decreased COL-1 expression in NOD dermal fibroblasts compared to that of control fibroblasts. Our findings suggest that impaired responsiveness of NOD fibroblasts to IFN-γ is not due to lack or dysfunction of cytokine receptor.

To further explore the mechanism underlying defective tryptophan catabolism in NOD dermal fibroblasts, we asked whether the protein machinery system of the cells is malfunctioning. Fibroblasts were transduced with IDO gene using adenoviral vector. In contrary to our observation after IFN-γ treatment, NOD fibroblasts were able to express IDO post transduction. This result proved that these cells are able to conduct transcription of the IDO gene and translate it to functional protein; we concluded that the defect in IFN-γ-mediated-tryptophan catabolism in NOD dermal fibroblasts is neither related to interaction of IFN-γ with its receptor nor gene transcription/translation mechanisms.

To get more insights into defective IDO expression in NOD fibroblasts, STAT1 phosphorylation was evaluated following IFN- γ treatment. We provided evidence that protein STAT1 does not get phosphorylated on tyrosine 701, impeding IFN-γ-induced- JAK/STAT1 pathway in NOD fibroblasts. This data was consistent with previous observation of tolerogenic DCs in early prediabetic NOD mice [9]. Thus, the blockade of JAK/STAT1 pathway in dermal fibroblasts from NOD mice is involved in a defect in IFN-γ induced-IDO expression.

Finally, we asked whether NOD dermal fibroblasts are able to express IDO through an IFN-γ-independent pathway. For this reason, we treated both C57BL/6 and NOD dermal fibroblasts with LPS. LPS has been shown to induce IDO expression in primary rat glial cultures without an increase in detectible IFN-γ [33], [34]. Although, it is widely accepted that IFN-γ is an essential factor for IDO induction through JAK/STAT1 pathway [25], [35], recent studies have revealed that IDO expression can be regulated by other inflammatory stimuli, including tumor necrosis factor-α and LPS [35]. LPS is shown to induce IDO expression through the activity of mitogen activated-protein kinase JNK. These data indicate that both IFN-γ-dependent and -independent pathways can mediate IDO expression. Our results showed that while IFN-γ fails to induce IDO expression in NOD dermal fibroblasts, these cells are able to express significant levels of active IDO enzyme in response to LPS treatment. Confirming the previous findings, our results showed that the LPS-induced IDO expression most likely occurs through JNK pathway. This finding suggests that fibroblasts are unable to produce IDO in response to pro-inflammatory cytokine i.e. IFN-γ, which is released from immune cells. However, these cells are competent to express IDO in the defense against bacterial infections, where LPS could play an important role in inducing this enzyme.

In conclusion, the findings of this study provide evidence that IFN-γ fails to induce IDO expression and tryptophan catabolism in dermal fibroblasts from either female or male NOD mice regardless of the stages of diabetes. Moreover, our data suggest that this defect is neither due to IFN-γ receptor dysfunction nor protein expression machinery of these cells. We found that impaired STAT1 phosphorylation might be partially responsible for this failure. Despite the defect in JAK/STAT1 pathway, we showed that other IFN-γ-mediated pathways like IFN-γ-induced MHC-I expression or IFN-γ-mediated inhibition of COL-I production were still functional. As stated before, both of these pathways can be activated independent from JAK/STAT1. Therefore, the observed defect in this pathway would not interfere with other aspects of IFN-γ-mediated gene expressions and functions. Although IFN-γ fails to induce tryptophan catabolism in NOD dermal fibroblasts, activation of an IFN-γ-independent pathway i.e. LPS-mediated JNK pathway can result in expression of active IDO enzyme.

## Materials and Methods

### Ethics Statement

This study has been approved by the University of British Columbia Animal Care Committee. All animals used in this study were maintained and undergone procedures in accordance with the principles of laboratory animal care and the guidelines of the University of British Columbia Animal Care Committee.

### Cell Culture and Treatments

Dermal fibroblasts were explanted from skin punch biopsies obtained from male C57BL/6 at 8 or 12 weeks of age, diabetic female NOD mice at 12 weeks of age, diabetic male NOD mice at 24 weeks of age, and male/female prediabetic NOD mice at 8 weeks of age (The Jackson Laboratories, Bar Harbor, ME, USA). As described previously [13], fibroblasts were cultured in Dulbecco’s Modified Eagle Medium (DMEM) (Invitrogen Life Technologies, Burlington, ON, Canada) supplemented by 10% fetal bovine serum (FBS) (Hyclone Laboratories, Inc, Logan, UT, USA), 100 U/ml penicillin, 100 µg/ml streptomycin and 0.25 µg/ml amphotericin B (Gibco, Invitrogen Incorporation, NY, USA), incubated in a humidified incubator at 37°C in 5% CO2. Cells at passage three to five were used for this study.

Splenic DCs were isolated from diabetic female NOD mice (>12 weeks of age) and fractioned based on CD11c expression using pluriBead® cell separation kit (pluriSelect GmbH, Leipzig, Germany) according to manufacturer’s instructions. Isolated DCs were cultured within RPMI-1640 (Hyclone Laboratories, Inc, Logan, UT, USA) medium supplemented by 10% FBS, 100 U/ml penicillin, 100 µg/ml streptomycin and 0.25 µg/ml amphotericin B in 6-well flat bottom culture plates (Corning Incorporated, Corning, NY, USA).

Fibroblasts were seeded on 6-well flat bottom cell culture plates and treated with IFN-γ (Sigma Chemicals, Oakville, ON, Canada) at different concentrations (0, 250, 500 or 1000 U/ml of DMEM plus 2% FBS) for either 24 hours (mRNA analysis) or 48 hours (protein analysis). Subsequently, CM of the cells were collected for Kyn assay and cell pellets were obtained for analyses of IDO, MHC-I and COL-I expressions. CD11c^ +^ DCs were treated with 1000 U IFN-γ per ml of RPMI-1640 medium plus 2% FBS or left untreated for 24 hours. Then, pellets of DCs were collected for evaluation of IDO mRNA expression.

To study IFN-γ-induced-STAT1 phosphorylation, cells were starved overnight and then treated with 1000 U/ml IFN-γ or left untreated in DMEM plus 2% FBS. Fibroblasts were harvested at different time points (15, 30 and 60 minutes after treatment) and cell pellets were collected for further analysis.

In an attempt to evaluate IDO expression via other IFN-γ-mediated pathways, LPS from *Pseudomonas aeruginosa* (Sigma, St. Louis, MO) was used to induce IDO expression in C57BL/6 and NOD dermal fibroblasts. Briefly, fibroblasts were seeded on 6-well flat bottom cell culture plates and treated with 1 µg/ml of LPS or left untreated in DMEM plus 2% FBS. After 24 hours, fibroblasts were harvested and subjected to mRNA isolation for IDO expression analysis. To confirm whether the LPS-induced IDO expression is mediated via JNK pathway, fibroblasts were treated with 1 µg/ml of LPS in the presence or absence of SP600125 (10 µM) (Sigma, St. Louis, MO), a JNK inhibitor or left untreated in DMEM plus 2% FBS for 48 hours. Subsequently, cell pellets were analyzed for IDO protein expression and CM were collected for Kyn assay.

### Transduction of IDO Gene in Cells Using Adenoviral Vector

Recombinant adenoviral vector carrying human IDO and GFP (Ad-IDO) was constructed as described before [36], and used to induce IDO expression in cells. Briefly, mouse dermal fibroblasts were seeded on 6-well flat bottom cell culture plates and were transfected with either Ad-IDO or a mock vector at 100 multiplicity of infection (MOI), or left untreated as control. Free viral particles were washed out after 30 hours. The expression of GFP was assessed using fluorescence microscopy (Nikone, Melville, N, H-1010 AF) to confirm successful IDO transduction.

### Kynurenine Assay

L-Kyn level was measured in CM of the treated cells as an indicator of IDO activity, as described previously [13]. Briefly, proteins were precipitated in CM samples using 30% trichloroacetic acid. Samples were centrifuged and afterward, 500 µl of supernatant from each sample was incubated with one volume of Ehrlich’s reagent (Sigma Chemicals, Oakville, ON, Canada). The absorbance of the solution was measured at 490 nm using spectrophotometry. Kyn levels were calculated using an equation obtained from standard curve.

### Western Blot Analyses of IDO and Collagen

IDO and COL-1 expressions were evaluated in mouse dermal fibroblasts by the previously established method [19]. Briefly, harvested cells were lysed in cell lysis buffer (containing 50 mM Tris-HCL, pH 7.4; 10 mM EDTA; 5 mM EGTA; 0.5% Nonidet P-40; 1% Triton X-100 and protease inhibitor cocktail (Sigma Incorporation, Oakville, ON, Canada)). After resolution on SDS-PAGE, proteins were transferred to PVDF membrane (Milipore Corp., Bedford, MA, USA). Membranes were blotted with polyclonal rabbit anti-human IDO antibody (1∶1000; Washington Biotechnology Inc, Baltimore, MD, USA) to detect IDO or monoclonal mouse anti-human COL-I antibody for collagen detection.

### Western Blot Analysis of STAT1 Phosphorylation

To evaluate the status of STAT1 phosphorylation in response to IFN-γ, cell pellets were lyzed in cell lysis buffer containing phosphatase inhibitor cocktail (Sigma Incorporation, Oakville, ON, Canada). Samples were run on SDS-PAGE and transferred to nitrocellulose membrane (Invitrogen, Carlsbad, CA, USA). Immunoblotting was performed using rabbit anti-mouse STAT1 antibody or rabbit anti-mouse phospho-STAT1 antibody (1∶1000; Cell Signaling Technology, Inc. Danvers, MA, USA) according to manufacturer’s protocol.

### Reverse-Transcriptase Analyses of IDO, MHC-I and Collagen

The total RNA in dermal fibroblasts was extracted using RNeasy kit (Qiagen, Maryland, USA) based on manufacturer’s instructions. Subsequently, cDNA was synthesized using SuperScript RT-PCR system (Invitrogen, Carlsbad, CA, USA). The primer’s used were as follows: IDO: sense 5′-GGCACACGCTATGGAAAACT-3′, antisense 5′-CGGACATCTCCATGACCTTT-3′ product size 296 bp; C57BL/6 mouse MHC-I (H-2b2): 5′-GCGAGGGTGGCTCTCACACG-3′, antisense 5′-TCAGGGTGAGGGGCTCAGGC-3′ product size 554 bp; NOD mouse MHC-I (H-2kd): sense 5′-GGGCGGCTCTCACACGTTCC-3′, antisense 5′-TCCCCTGCAGGCCTGGTCTC-3′; mouse COL-I (Col 1a1): sense 5′-GACGCCATCAAGGTCTACTG-3′, antisense 5′-ACGGGAATCCATCGGTCA-3′, product size 154 bp; mouse glyceraldehyde-3-phosphate dehydrogenase (GAPDH): sense 5′-TGGCACAGTCAAGGCTGAGA-3, antisense 5′-CTTCTGAGTGGCAGTGATGG-3′ product size 384 bp; mouse β-actin: sense 5′–GTGGGCCGCCCTAGGCACCAA-3′, antisense 5′–CTCTTTGATGTCACGCACGATTTC-3′. GAPDH and β-actin mRNA levels were used as internal controls. RT-PCR cycles were optimized for each set of primers. The numbers of cycles were as follows: 42 cycles for IDO, 38 cycles for COL-1 and 38 cycles for MHC-I. Amplified RT-PCR products were separated by electrophoresis on 1% agarose gel.

### Statistical Analysis

Data are reported as Mean±SEM. The statistical analysis was performed by one-way ANOVA followed by post hoc evaluation using student’s t test for multiple comparisons to ensure the proper distribution of variances. P-value of less than 0.05 was considered statistically significant.
